# First Human Isolate of Hantavirus (*Andes virus*) in the Americas

**DOI:** 10.3201/eid0807.010277

**Published:** 2002-07

**Authors:** Hector Galeno, Judith Mora, Eliecer Villagra, Jorge Fernandez, Jury Hernandez, Gregory J. Mertz, Eugenio Ramirez

**Affiliations:** * Public Health Institute of Chile, Santiago, Chile; † Los Angeles Hospital, Santiago, Chile; ‡ University of New Mexico Health Sciences Center, Albuquerque, New Mexico, USA

**Keywords:** Hantavirus, Andes virus, Hantavirus Pulmonary Syndrome

## Abstract

We isolated Andes virus (formal name: *Andes virus* [ANDV], a species in the genus *Hantavirus*)*,* from serum of an asymptomatic 10-year-old Chilean boy who died 6 days later of hantavirus pulmonary syndrome (HPS). The serum was obtained 12 days after his grandmother died from HPS and 2 days before he became febrile. No hantavirus immunoglobulin (Ig) G or IgM antibodies were detected in the serum sample. After three blind passages, ANDV antigens were detected in Vero E6 cells by immunofluorescence assay and enzyme-linked immunosorbent assay, and ANDV RNA was detected by reverse transcription-polymerase chain reaction. A fragment of the virus genome showed 96.2% nucleotide identity with that of prototype ANDV. To our knowledge, this is the first isolation of any agent of hemorrhagic fever with renal syndrome from a human and the first such isolation of hantavirus before symptoms of that syndrome or HPS began.

 Hantaviruses are rodent-borne negative-sense RNA viruses that cause hemorrhagic fever with renal syndrome or hantavirus pulmonary syndrome (HPS) in humans (1). HPS is caused by New World hantaviruses that have been identified since the syndrome was first recognized in the southwestern United States in 1993 (2). The syndrome is characterized by four stages: the febrile prodrome, the cardiopulmonary stage, diuresis, and convalescence. The cardiopulmonary phase, which typically lasts 2–10 days, can range from a mild illness, characterized by shortness of breath and need for supplemental nasal oxygen, to severe cardiopulmonary involvement with respiratory failure, lactic acidosis, shock, and death (3,4). In survivors, diuresis occurs abruptly and is usually associated with rapid clinical improvement. The course and duration of the convalescent stage are more variable, but most patients describe persistent fatigue and limited tolerance for exercise for at least several months.

 HPS has been reported in Argentina, Brazil, Canada, Chile, Panama, Paraguay, Uruguay, and the United States; mortality rates range from 40% to 50% (1). In Chile, 135 cases of HPS have been reported through February 9, 2001, with a 48.8% mortality rate (5). Andes virus (formal name: *Andes virus* [ANDV], a species in the genus *Hantavirus),* which is carried by *Oligoryzomys longicaudatus,* is responsible for most HPS cases in Argentina and Chile. In contrast, Sin Nombre virus (formal name: *Sin Nombre virus* [SNV]), which is carried by *Peromyscus maniculatus,* is the primary pathogen in North America. No evidence has been found to support person-to-person transmission of SNV, but person-to-person transmission of ANDV has been documented in one large outbreak in Argentina (6) and is suggested by case clustering in household contacts in Chile (M. Ferres, X. Aguilera, pers. comm.).

 Most patients are seen at the onset of the cardiopulmonary phase, and information about clinical and laboratory findings, viremia, and immune responses is most complete for this and subsequent phases (7,8). Less is known about clinical and laboratory findings, viremia, and immune responses during the febrile prodrome, although both specific immunoglobulin (Ig) G and IgM antibodies are almost always present during this phase (9). In contrast, no information is available on the development or time course of viremia or immune responses before symptoms begin (in the prodromal phase). We describe the first isolation of hantavirus from a human in the Americas and the first isolation of hantavirus from a human before onset of symptoms of HPS or hemorrhagic fever with renal syndrome.

## Patients and Methods

### Case Descriptions

 The index patient was a 54-year-old woman who had headache, myalgias, and abdominal pain on August 26, 1999, followed several days later by respiratory symptoms. She went to the hospital on August 31, where she was diagnosed with bilateral pneumonia and adult respiratory distress syndrome; she died on September 1. A serum sample obtained on August 31 was reactive for IgM antibodies. The patient’s 71-year-old brother had had a febrile illness on August 7, 1999, and was hospitalized 2 days later with a clinical diagnosis of acute abdominal pain**,** pyelonephritis, shock, and bilateral pulmonary infiltrates; he died on August 10. HPS was not suspected, and no serum or tissue was available for testing when HPS was diagnosed in the index patient.

 The Ministry of Health initiated a routine evaluation of household and neighborhood contacts on September 13, 1999. Blood was obtained from 10 asymptomatic contacts, including the 10-year-old grandson of the index patient. On September 15, the grandson became febrile, and headache and vomiting developed. Two days later, he (patient 99-7913) was evaluated as an outpatient. His physical examination showed fever (38°C) and no respiratory symptoms***.*** His leukocyte count was 13,000/µL, hematocrit 46.9%, hemoglobin 15.7 g/dL, and platelet count 125,000/µL. The plasma C reactive protein was 39 mg/L. Diffuse bilateral interstitial pulmonary infiltrates were detected on chest radiograms, and the patient was treated with a macrolide antibiotic for presumed pneumonia. He returned to the hospital the morning of September 18 without fever, with arterial pressure 110/60 mmHg, tachycardia (100 beats per minute), and weakness. Pneumonia, obstructive bronchial syndrome, and dehydration were diagnosed. He was treated with intravenous penicillin, hydration, and aerosolized salbutamol. He returned to the hospital again on the evening of September 18 with respiratory failure and shock and died on September 19 within hours of arrival. No additional serum or tissue samples were obtained at the outpatient visit or in the hospital.

### Epidemiologic Studies

 Routine epidemiologic evaluation of each confirmed HPS case in Chile includes rodent trapping around the patient’s household and evaluation of household and family contacts. The latter includes a clinical evaluation for history of recent fever or other symptoms and the administration of a questionnaire to assess risk factors for hantavirus infection. A serum sample is obtained from household and family contacts by venipuncture and transported to the Institute of Public Health in Santiago for determination of hantavirus antibodies.

### Biosafety Procedures

 We followed the recommendations of the Centers for Disease Control and Prevention (CDC) in all aspects of this work (10). Antibody studies were conducted at biosafety level 2 (BSL-2) facilities, but viral isolation attempts were conducted at BSL-3 laboratories in the Instituto de Salud Pública laboratory in Santiago.

### Detection of Antibodies to SNV in Patient Serum

We detected antibodies from patient serum samples by enzyme-linked immunosorbent assay (ELISA) with nucleocapsid (N) antigens of SNV and Laguna Negra (formal name: *Laguna Negra virus* [LNV]) virus. These diagnostic tests were obtained from CDC in Atlanta, Georgia (11). SNV antigen was used in the solid phase for detecting IgG antibodies, and LNV antigen was used in an antigen-capture format for detecting specific IgM antibodies, as described (11).

### Isolation of ANDV from Patient Serum

 Virus was isolated by a conventional method, with three blind passages in monolayers of Vero E6 cells (12). We grew Vero E6 cells to confluence in T25 flasks with minimal essential media containing 10% fetal calf serum and antibiotics, and then replaced the media with 1.5 mL of fresh media containing 0.5 mL of serum from patient 99-7913. After 1 hour, we added 4.5 mL of fresh media and maintained the cells at 37°C in 5% CO2 for 26 days, refeeding twice per week (Tissue Culture 1 or TC-1). At 26 days, the cells were treated with trypsin and split 1:2 into fresh T25 flasks (TC-2). At 24 days postinoculation (dpi), we trypsinized the cells and replated them into fresh T25 flasks (TC-3). At 14 dpi, we trypsinized the cells and replated them into fresh T25 flasks (TC-4). At 13 dpi, we treated the cells with trypsin and replated them after removing 5 x10^4^ for indirect immunofluorescence assay (IFA) analysis.

### IFA

 Fifty thousand inoculated Vero E6 cells were washed twice with phosphate-buffered saline and dried on a microscope spot-slide for IFA testing. As a control, we used uninoculated Vero E6 cells that were processed in parallel. The cells were stained according to Gallo et al. (13), using a 1:1,000 dilution of rabbit polyclonal anti-Andes N. Cells were considered to be positive for hantavirus antigen if we observed punctate cytoplasmic and Golgi staining in the presence of anti-N antibody but not in the presence of preimmune rabbit serum or if the cells had not been injected with a source of ANDV.

### Reverse Transcription-Polymerase Chain Reaction (RT-PCR)

 We conducted nested RT-PCR to detect a portion of the viral N gene by using generic primers for S segment as described (14). The coordinates of the primers, designated S1 and S2 (outer primers), were at 2 and 593, whereas the inner primers S3 and S4 were at coordinates 22 and 359. We determined that an amplification reaction was positive if we could visualize a 338-bp band after electrophoresis through agarose, as described (14).

### Phylogenetic Studies

 Nucleotide sequences examined corresponded to positions 22–359 of antigenome-sense sequence of nucleoprotein gene. Genetic relationships of the Chilean isolate with homologous sequences of previously characterized hantavirus were obtained by the maximun parsimony method with the Clustal W and PHYLIP packages (15).

## Results

 The grandson’s death from suspected HPS was reported on September 21. Serum obtained on September 13, which had been transported and stored at 4°C, was tested for the presence of hantavirus antibodies by ELISA. Neither IgG nor IgM antibody was detected. Serum was then injected into tissue Vero E6 cell monolayers to attempt viral isolation (12).

### Virus Isolation

**Figure 1:**
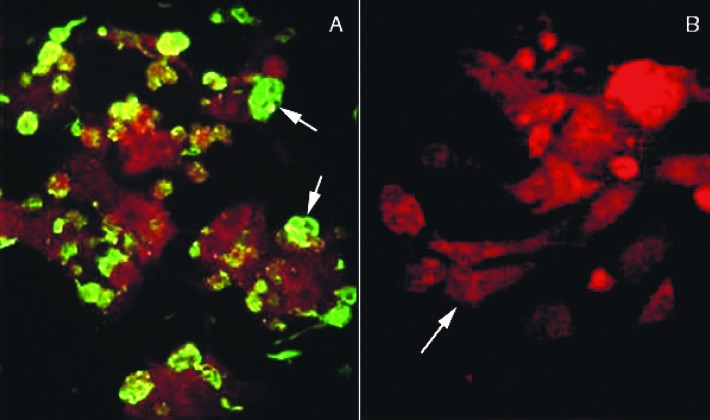
Immunofluorescence assay (IFA) of Vero E6 cells infected with Chilean hantavirus CHI-7913 isolate. A, IFA with seropositive human sera from a Chilean HPS patient; arrow shows infected Vero E6 cells expressing hantavirus antigens. B, IFA with seronegative human sera from uninfected control; arrow shows the negative IFA of Vero E6 cells infected with the CHI-7913 isolate.

 Inoculated and noninoculated Vero E6 cells were cultivated for several weeks. After three blind passages in new monolayers of Vero E6 cells, the presence of hantaviral antigens was tested by IFA with serum samples from seropositive Chilean HPS patients. Only the cells derived from the patient serum-inoculated tissue culture expressed fluorescent inclusion bodies (Figure 1). Over 90% of the cells became fluorescent. The specificity of this reaction was further tested by ELISA with cellular lysates prepared from infected and noninfected cells and ANDV antibody-positive rabbit sera. The absorbance values obtained by using lysates of infected cells were higher than those from noninfected cells (data not shown). To further confirm the presence of hantavirus infection in the Vero E6 cells, we also used nested RT-PCR of the nucleoprotein gene to test tissue cultures inoculated with patient serum with generic primers for S segment (14). Only the RNA extract from patient sera-inoculated cells showed an amplified product with the expected 338-bp fragment. The amplified product was not detected in mock-infected cells (data not shown).

**Figure 2:**
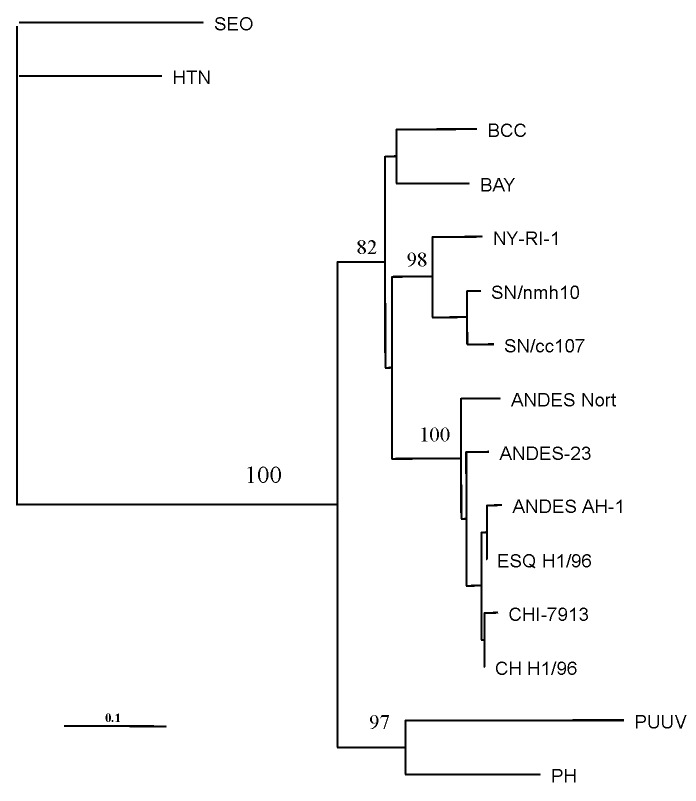
Maximum-parsimony tree analysis comparing S nucleotide sequence of CHI-7913 virus with homologous sequences of previously characterized hantaviruses. Nucleotide sequences examined correspond to positions 22–359 of antigenome-sense sequence of nucleoprotein (N) gene. Sequences were analyzed by the maximun parsimony method with the Clustal W and PHYLIP packages (15). The minimal length trees shown were supported as the majority rule consensus tree in 500 replicates. The bootstrap replicates supporting each node are indicated. References and GenBank accession numbers for the sequences used in S segment comparisons are BCC (16) L39949; BAY (17) L36929; NY strain RI-1 (18), U09488; SN strain cc107 (19), L33683; SN strain nmh10 (20), L25784; PH strain PH-1 (21), and M34011; Puumala strains Sotkamo (22), X61035; Seoul (SEO) strain sr-11 (23), and M34881; Hantaan (HTN) strain 76-118 (24), M14626; Andes strain AH-1 (14), AF004660; ESQ H-1/96 (14), AF005948; CH H-1/96 (14), AF 005947; AND Nort (strain unpublished) AF325966; and Andes strain 23 (AF291702).

 The amplified DNA product was sequenced and compared with the sequence of prototype strains (Figure 2). The Chilean hantavirus isolate, designated CHI-7913, showed 96.2% nucleotide identity with the prototypical ANDV sequence, but only 81.1%–82.5% identity with SNV from the southwestern United States (14).

## Discussion

 Previous human isolates of hantaviruses have been reported only for Old World viral species, and no isolates have been described from patients who were asymptomatic. Since previous attempts at other laboratories had been unsuccessful, we attempted isolation before the onset of symptoms, and our first attempt to do so was successful. Our results document the presence of infectious hantaviral virions in a serum specimen obtained from a seronegative 10-year-old child 2 days before his symptoms began and 6 days before his death from HPS. We excluded the possibility of laboratory contamination since no hantavirus was or had been in use in our laboratory nor were we making other attempts to isolate hantavirus.

 Based on the partial S segment sequence we obtained, the isolate CHI-7913 is a geographic variant of ANDV. Comparison of CHI-7913 virus N gene sequence with the corresponding sequences of representative New World hantaviruses showed the highest degree of identity with that of ANDV.

 Isolation of hantaviruses from rodents and humans has been difficult, and isolation from humans has been particularly so. Many apparent human isolates were obtained in laboratories that were actively cultivating a number of hantavirus strains at the time of the isolation. Thus, several earlier human isolates have proven difficult to confirm as independent isolates after they were subjected to genetic comparison with previously obtained rodent isolates (25). Other human isolates that have been reported more recently have not been subjected to similar scrutiny. In 19 attempts over more than a decade, Juto et al. reported one successful attempt at isolation of Puumala virus (formal name: *Puumala virus* [PUUV]) from phytohemagglutinin-stimulated human leukocytes (26). In a report of isolation of Hantaan virus (formal name: *Hantaan virus* [HTNV]) from peritoneal exudate cells collected from a patient with severe hemorrhagic fever with renal syndrome on the 10th day of illness, Gu et al. noted that human isolation of HTNV is easier from blood collected during the first 4 days of illness than from blood collected after day 6 (27). Examining these and other human isolates of PUUV and HTNV by sequence analysis will be valuable in confirming their independent origin, much as we have been able to do with CH-7913.

 We suspect that development of neutralizing antibody early in symptomatic illness may be the primary factor leading to difficulty in isolation of hantavirus from blood in humans after illness has begun. Bharadwaj et al. recently reported detecting neutralizing antibody in all sera obtained at the day of hospital admission in patients with SNV-associated HPS (9). Although most hospital admissions occurred at the onset of the cardiopulmonary stage, neutralizing antibody was also detected in a limited number of sera available 1 or 2 days before hospital admission, during the prodromal phase. The recent report that viral RNA detected by RT-PCR inevitably declines early in hospitalization (7,28) is also consistent with the hypothesis that virus is present but that neutralizing antibody and other immune responses are already reducing the titer and infectiousness of hantaviruses by the cardiopulmonary stage of illness. That stage is when most patients come to medical attention.

 We were able to isolate hantavirus from serum obtained 2 days before symptoms began and before the production of detectable levels of IgG or IgM antibodies. This finding suggests that a viremic phase may precede symptoms and that the onset of symptoms in the prodromal stage may be associated with humoral and cellular immune responses rather than viremia. In contrast to HPS in North America, where case clusters are uncommon, approximately one third of HPS cases in Chile have occurred in clusters involving members of the same household or other close contacts (M Ferres, X Aguilera, pers. comm.). Of these, most have involved case clusters with symptom onset separated by 2–4 weeks rather than case clusters with closely related dates of symptom onset. As such, Chile may pose a unique opportunity to prospectively follow close contacts of index patients to determine whether viremia routinely precedes symptoms as well as to identify and perhaps treat some persons early in the course of symptomatic hantavirus illness.
